# The Angel in the Marble: Antibiotic Duration in the Neonatal Intensive Care Unit

**DOI:** 10.3390/antibiotics15020228

**Published:** 2026-02-20

**Authors:** Joseph B. Cantey, Dalyn B. Guinn

**Affiliations:** 1 Department of Pediatrics, Divisions of Neonatology and Immunology & Infectious Diseases, University of Texas Health San Antonio, 7703 Floyd Curl Dr, San Antonio, TX 78232, USA; 2 Department of Pediatrics, Division of Neonatology, University of Texas Health San Antonio, 7703 Floyd Curl Dr, San Antonio, TX 78232, USA; guinnd@uthscsa.edu

**Keywords:** antibiotic, meningitis, necrotizing enterocolitis, neonate, sepsis, stewardship

## Abstract

Antimicrobial stewardship in the neonatal intensive care unit is a critically important tool to optimize clinical outcomes. The ideal duration of antimicrobial treatment is a key area that contains many knowledge gaps. This narrative review has three aims. One, to highlight the existing evidence for empiric and definitive antibiotic treatment durations for infants; two, to focus on clinical situations where further studies are needed; and three, to propose a rational, goal-based approach to clinical studies that provide for infant safety as shorter treatment durations are investigated.

## 1. Introduction

“*Ho visto un angelo nel marmo ed ho scolpito fino a liberarlo*.” I saw the angel in the marble and carved until it was free. This oft-quoted saying, attributed to the sculptor Michelangelo [1], is an ideal metaphor for antimicrobial stewardship. In the neonatal intensive care unit (NICU), as in other clinical settings, the goal of stewardship is not to reduce antibiotic utilization to zero. If it were, we could achieve zero-use tomorrow—provided we were prepared to accept the cataclysmic decline in infants’ clinical outcomes. Sufficient antimicrobial therapy to resolve an infection and prevent its recurrence is critical; if treatment duration is left too short, morbidity and mortality from neonatal sepsis, meningitis, and necrotizing enterocolitis (NEC) would skyrocket. Conversely, NICU providers cannot use antibiotics indiscriminately without consideration of the harm that follows. These well-documented adverse outcomes are directly linked to duration of therapy and include short-term (drug toxicity, antimicrobial resistance, dysbiosis), medium-term (increased rates of sepsis, NEC, death) and long-term complications (metabolic syndrome, obesity, autoimmune diseases) [2,3,4,5]. We must instead approach the duration of antimicrobial treatment in the manner of Goldilocks—not too little, not too much, but just right [6].

Unfortunately, “just right” can be a challenge. “Just right” must be driven by clinical experience mixed with targeted research. NICU stewardship is as much an art as it is a science; like Michelangelo, we must strive to chisel away the prolonged and unnecessary antibiotic use, until only the necessary remains. Remove too much, and the sculpture is ruined; remove too little, and there is work left to do. Of course, “I saw the angel in the marble” may be apocryphal; many quotes from Michelangelo (or Twain, or Aristotle) are. If you prefer, we can rely on another of his quotes that we can be certain of, one that serves our purposes just as well. Michelangelo, writing to Benedetto Varchi in 1552, remarked, “The sculptor arrives at his end by taking away what is superfluous.” [7]. But what, exactly, is superfluous antibiotic use among neonates? How do we separate the necessary from the unnecessary and, most importantly, how do we do it safely? This narrative review is intended to highlight what is known regarding optimal antibiotic duration for empiric and targeted treatment in the NICU setting, and to provide a framework for future clinical research in this field. Let us see if we cannot free the angel.

## 2. Methods

To prepare this narrative review, the authors searched the National Library of Medicine (PubMed) using the terms “antibiotic duration” and “antibiotic” exploded, along with one of “newborn”, “neonate”, or “neonatal intensive care unit”, filtered by “human” and English language, from 1970 to the present. This search resulted in 1758 articles. Quality improvement studies were excluded. A second search was then performed with “antibiotic duration” and “neonatal intensive care unit” without filters to ensure that no germane articles were missed; this search resulted in 430 articles with near-complete overlap. Articles titles and relevant abstracts were reviewed for observational studies or clinical trials with an emphasis on treatment duration. This review is meant to be illustrative rather than completely comprehensive; as such, the authors aimed to avoid redundancy in review articles but did include all clinical trials with a focus on duration of therapy. For the purposes of this review, “optimal duration” is defined as the duration that eradicates acute infection, prevents recurrence of the infection, and minimizes adverse outcomes of antibiotic exposure.

## 3. Empiric Therapy

Empiric therapy includes all antibiotics administered to neonates for 24–48 h while awaiting the results of culture, molecular testing, or other diagnostic assays intended to determine whether infection is present. Empiric antibiotics, or “rule-out” courses, represent the bulk of antibiotic use in the NICU setting. [8] Therefore, although rule-out courses are generally brief, even small reductions in their duration can have major impacts on total antibiotic consumption within a given NICU.

Historically, rule-out sepsis courses were 48–72 h and could be extended inadvertently if the discontinuation needed to be an active decision—that is, one made during daily rounds. In the last ten to fifteen years, hard-stops, where the antibiotics are set to discontinue automatically by the electronic medical record, have proven to be safe and effective at limiting the antibiotic course to the intended duration and are increasingly used. In addition, centers have evaluated shorter rule-out courses, first 36 h and now, in some cases, as short as 24 h [9,10]. The primary consideration is ensuring that antibiotic coverage continues until cultures have incubated for long enough for clinicians to be confident of their sterility. Limiting early antibiotic exposure to 24–36 h provides time for cultures to grow; approximately 90–95% of blood cultures will be positive by 24 h of incubation and virtually all by 36 h [11,12].

However, there are two considerations that temper enthusiasm for further reduction in the duration of empiric therapy. The first is the aforementioned time to positivity of cultures. At present, there are no non-culture-based modalities (i.e., biomarkers) with sufficient sensitivity or specificity to forego obtaining a culture [13,14]. Therefore, durations shorter than 24 h may be insufficient if blood cultures are still incubating; no neonatologist wants to discontinue therapy at 12 h only to discover *E. coli* in the infant’s culture at 18 h of life. Urine, cerebrospinal fluid, and other body fluids may take even longer to result; although their incubation times in vitro are approximately the same (e.g., the majority of cultures being positive within 24–36 h) [15,16,17]. In most microbiology labs, those plates are read manually at discrete times during the day rather than continuously like automated blood cultures. The use of molecular assays, particularly for cerebrospinal fluid, has significantly reduced the time to diagnosis in infants with infections detectable by current molecular panels, but those assays still rely on culture positivity rather than direct specimen application [18]. Until an optimal biomarker or molecular test can shorten the diagnostic window to minutes rather than hours, empiric antibiotics for a minimum of 24 h will remain necessary.

The second consideration for duration of empiric therapy is that there is some evidence—at present, only very exploratory evidence—to support potential benefit from a short empiric antibiotic course among preterm infants [19]. Prolonged early antibiotics are clearly associated with gut dysbiosis—specifically, a loss of diversity in the gut microbiome with predominance of pathogenic Gram-negative organisms—but a short course of early antibiotics could, in theory, be protective. Limited evidence suggests that a short course of early oral antibiotics can reduce the risk of NEC, but this benefit may be offset by a sharp increase in antibiotic-resistant organisms [20]. In theory, infants born with an abnormal microbiome—a dysbiosis inherited vertically following maternal antibiotic administration—could benefit from a “reset” of their microflora during a critical early window. Antibiotics may delay bacterial colonization, allowing gut maturation and sharpened innate immunity before the arrival of pathogenic bacteria [19]. However, data are currently limited to oral therapy, whether parenteral antibiotics provide this “reset” is unclear.

Current evidence, both in animal models and in observational studies of preterm infants, support that among infants who do not have early-onset sepsis (i.e., positive blood culture in the first 72 h of life), a short course of antibiotics (24–48 h) is associated with better clinical outcomes than a prolonged course (≥5 days) [2,3,4]. The benefits of avoiding antibiotics altogether are more difficult to elucidate, primarily because observational studies are plagued by confounders, including confounding by indication—only very healthy preterm infants can avoid antibiotics entirely—survival bias, and heterogeneous practice between centers. These confounders emphasize the lack of randomized controlled trials for empiric therapy. The NICU Antibiotics and Outcomes (NANO) trial aimed to address this knowledge gap [21]. The NANO trial enrolled moderate-risk preterm infants 23–30 weeks gestation without early-onset sepsis or gut perforation. These infants were randomized to 36–72 h of early antibiotics versus placebo. The primary outcome was a composite of death, late-onset sepsis (i.e., sepsis after 72 h of life), or NEC; microbiome data were also collected. At the time of this article, the NANO study had completed but analysis was ongoing. The NANO study will provide important information regarding the benefits or harms of a short course of early antibiotics compared to no antibiotics at all.

## 4. Definitive Therapy

**Figure 1:**
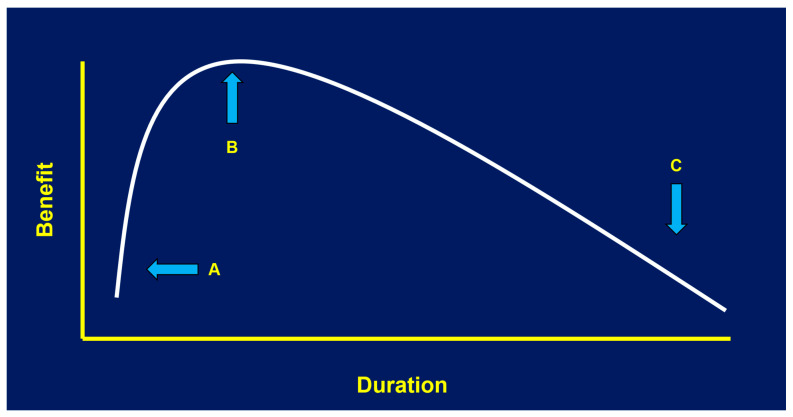
The hypothetical benefits of antimicrobial therapy for a given infection are shown on the *y*-axis. If the duration of therapy is too short (A), the full benefits of treatment are not yet realized. However, if duration of therapy is too long (C), adverse effects of antimicrobial exposure accumulate and begin to reduce the benefit of treatment. Optimal duration (B) is when maximum benefit has been achieved (e.g., clinical cure, minimal risk of recurrence) but before prolonged or unnecessary therapy begins.

When infection is definitively ruled in, or at least strongly suspected by the clinician based on specific clinical signs, one might expect that treatment durations would become more evidence-based than with empiric therapy. Surprisingly, this is not the case. Treatment durations for common neonatal infections are usually more dogmatic than scientific, as there is a remarkable paucity of randomized clinical trials focused on specific treatment duration for different neonatal infections. Predictably, opinion and institutional norms fill the void created by a lack of evidence, which can lead to significant practice variation within and between centers. Hopefully, we can agree that the goals of treatment are threefold. First, to eradicate acute infection (e.g., clinical improvement, sterilization of culture or normalization of molecular tests, and when appropriate, improvement of laboratory values or radiographic findings); second, to prevent recurrence of the infection in the short term; and third, to stop antimicrobial therapy in time to minimize adverse outcomes of antibiotic exposure (Figure 1). To satisfy the third criterion, we must strive to find the shortest treatment duration that satisfies the first two objectives; this duration *should* be the optimal duration.

Step one is to resolve the acute infection. Most often, this is a culture-based measurement. Most bloodstream infections among neonates resolve quickly following administration of effective therapy; the median is 1 day of positive cultures [22]. For the minority of cultures that are persistently positive, either ineffective antibiotic selection or lack of source control are usually the issues. For urinary tract infections (UTIs), clearance is usually within 24 h. Repeat urine culture as a “test of cure” is not generally recommended under normal circumstances, although complications such as indwelling hardware or failure to clinically respond to treatment may prompt clinicians to resample. Meningitis should begin to resolve within 48 h; persistence of organism in culture after 48 h of therapy is a poor prognostic indicator [23]. For conditions where cultures are not the reference standard, clear clinical resolution may take longer. For example, radiographic findings in neonatal pneumonia lag behind clinical improvement and microbiologic clearance, often by weeks [24]. Similarly, the value of follow-up imaging for NEC after treatment is suboptimal and has been shown to be inferior to normalization of clinical findings (e.g., feeding tolerance, resolution of abdominal distention) [25]. Molecular modalities may lag as well; the incredible sensitivity of DNA-based assays can lead to persistent positive test results long after cultures have cleared; whether this is a feature or a bug (no pun intended) is a source of contention.

Bloodstream infection. Step two is to prevent the infection from recurring promptly when antibiotics are discontinued. Observational studies have defined recurrence differently, but in general, repeat infection with the same organism within 10–21 days is generally considered a recurrence. Kempley et al. [26] found no association between treatment duration for *Staphylococcus aureus* bacteremia (5 days to >1 month) and risk for recurrence. Similarly, Djordjevich et al. [27] found no association between treatment duration (7–28 days) and recurrence among 76 neonates with Gram-negative sepsis; both treatment failures were treated for >14 days and were both due to a retained central venous catheter. Other observational studies have consistently shown that shorter courses are non-inferior to longer courses for *S. aureus* sepsis [28]. All of these observational studies are subject to significant cofounding, however, and should be considered hypothesis-generating at best.

In terms of randomized controlled trials, Chowdhary et al. [29] randomized 69 term and late preterm infants to 7 versus 14 days for bloodstream infection and found no difference in treatment failure or recurrence risk; the 2 treatment failures in the short-duration group were methicillin-susceptible *S. aureus* due to failure to remove a central venous catheter; the treatment failure in the long-duration group was *Klebsiella pneumoniae* and was not line-associated. A similar randomized trial by Dutta et al. [30] found no differences between 7 and 14 days of treatment also showed no differences in outcome, although long-term safety measures trended in support of shorter duration. For Rohatgi et al. [31] randomized 128 neonates with bloodstream infections to 7 versus 10 days of therapy and did not see a difference in treatment failure or recurrence. Other studies have randomized infants with proven sepsis to 10 versus 14 days with no differences in treatment failure or recurrence, although there was an increase in central venous catheter-associated complications in the longer therapy groups [32,33]. These three studies are a perfect illustration of the task that faces providers—7 vs. 10 vs. 14 days in a true round-robin competition, with no obvious differences in outcomes. Which, then, is best?

Urinary tract infection. UTIs have been perhaps better studied in terms of duration, but unfortunately, the focus has been on duration of intravenous therapy rather than total duration of antibiotics. Multiple randomized controlled trials and observational studies have shown no association between the duration of intravenous therapy before oral conversion and risk of treatment failure or recurrence. Marsh et al. [34] found no association between total duration of therapy and treatment failure; only 1 of 112 neonates in that cohort had treatment failure; that infant received 14 days of therapy (3 intravenous, 11 oral) and had recurrent UTI with the same organism—*K. pneumoniae*—within 1 week of completing antibiotics, despite normal renal ultrasound and voiding cystourethrogram. At present, there are no randomized controlled trials of total duration of therapy for UTI in the NICU setting. This remains one of the largest knowledge gaps in neonatal antimicrobial stewardship.

Meningitis. The optimal duration of therapy for neonatal meningitis has also not been well studied. Given its combination of rarity and severity, it is unsurprising that prolonged treatment durations are the textbook standard for meningitis, particularly among preterm infants. For Gram-positive organisms (e.g., group B *Streptococcus*), a minimum of 14 days is generally recommended; for Gram-negative organisms, this is commonly extended to 21 days or longer [35]. Suppurative complications, such as subdural empyema, brain abscesses, or ventriculitis, may extend therapy even longer [36]. Imaging studies such as contrast-enhanced brain MRI may be slow to normalize, further increasing treatment duration. Randomized controlled trials have generally excluded neonates. Molyneux et al. [37] investigated 1004 children age 2 months—12 years with pneumococcal, meningococcal, or *Haemophilus influenzae* type B meningitis. Children who were doing well after 5 days of ceftriaxone therapy were then randomized to 5 or 10 total days of ceftriaxone with no differences observed in outcomes or recurrence risk. However, readers will—correctly—point out that these are neither neonates nor neonatal pathogens, so the generalizability of this study to the NICU setting is suspect. NICU-specific data is limited; Mathur et al. [38] randomized infants with meningitis to 10 versus 14 days of therapy but had unacceptably high rates of treatment failure even in the 14-day group (14.3%). It is doubtful that anyone would describe meningitis treatment duration as “low-hanging fruit” in neonatal stewardship research.

*Culture-negative sepsis*. Treatment for suspected but not culture-proven sepsis, or “culture-negative” sepsis, accounts for a significant proportion of antimicrobial use in the neonatal intensive care unit [8]. “Culture-negative” sepsis is generally defined as clinical suspicion for infection due to specific clinical signs consistent with (but not necessarily specific for) infection, but without microbiologic identification of a pathogen despite reasonable testing. Such infants will receive empiric antibiotic therapy for 24–48 h while cultures are pending. Infants may receive prolonged therapy if their clinical signs persist, but the degree to which they benefit from additional therapy is unclear. Frameworks exist for the management of culture-negative sepsis in the NICU setting [39]. However, data regarding the optimal duration of therapy is limited. There is no evidence supporting improved outcomes with prolonged therapy; single-center observational studies have suggested good clinical outcomes with durations as short as 5 days [40,41]. Additional studies are needed to determine if 2–3 days of therapy are equally effective as 5 days.

*Necrotizing enterocolitis*. There are currently no randomized controlled trials regarding the optimal duration of NEC therapy. A few trials have investigated antibiotic selection and suggest modest benefits with the addition of anaerobic therapy, which is biologically plausible given the abundance of anaerobic bacteria in the intestinal tract [42,43]. None, however, have compared different durations of therapy head-to-head. There are—somehow—more randomized controlled trials on the surgical management of NEC than there are on the duration of antimicrobial therapy [44,45]. In the absence of clinical trials, the majority of neonatologists rely on general consensus or institutional protocols to guide duration of therapy. Commonly quoted durations in surveys are 36–72 h for NEC stage I (i.e., NEC rule-outs), 5–10 days for NEC stage II (i.e., medical NEC), and 10–14 days or more for NEC stage III (i.e., surgical NEC) [46,47]. However, observational studies have not associated treatment duration with either outcome or recurrence risk, nor have observational studies shown that length of treatment or broader-spectrum treatment can modify the risk for intestinal perforation [48,49]. Given the relative frequency and potential severity of NEC in NICUs worldwide, a randomized controlled trial investigating optimal duration of therapy for NEC stage II and III is long overdue.

*Pneumonia*. As mentioned above, the goal of optimizing treatment duration is to find the sweet spot of “long enough.” Perhaps the best success of this approach has been in neonatal pneumonia. This is despite—or perhaps because of—the lack of an objective diagnostic gold standard for this infection. Neonatal pneumonia is challenging to diagnose for a few reasons. First, most infants with pneumonia have sterile blood cultures. Second, culture from the respiratory tract is generally from the non-sterile upper airway, which makes pathogen identification not so much finding the needle in the haystack as much as deciding which needle looks suspicious in a whole pile of needles. Lower respiratory tract cultures (e.g., bronchoscopy) are not easily obtained in neonates. Finally, neonates have a tremendous number of non-infectious causes of respiratory distress and abnormal chest radiographs, many of which can mimic pneumonia. Therefore, most clinical research into neonatal pneumonia starts with a degree of classification bias. There are two randomized controlled trials that have investigated 4 versus 7 days for term or late-preterm infants with pneumonia; both studies showed universally good outcomes in both arms [50,51]. However, a follow-up study from one of those groups that investigated 2 versus 4 days found that 25% of infants failed the 2-day arm, compared to no treatment failures in the 4-day arm [52]. This is an excellent example, albeit with a relatively small sample size, of cautiously identifying a duration that is demonstrably too short and defining a lower boundary for safe treatment duration.

It is important to note that risk tolerance has a significant impact on current clinical management and future investigation of these clinical situations. Clinicians and researchers alike should have less appetite for risk with truly life-threatening infections (e.g., bloodstream infection, meningitis, NEC with gut perforation) than with less severe infections (e.g., UTI, pneumonia, suspected sepsis or lower-stage NEC). Both clinical management and research must be beholden to patient safety first and foremost.

## 5. Framework for Optimal Duration Research

How, then, do we safely investigate the optimal duration for a given proven or suspected infection among neonates? There are several common themes that should serve as the foundation for such studies; they are summarized below (Table 1). This framework can help guide future research in this critical area of neonatology.

First, we authors believe that non-inferiority of shorter durations of antimicrobials is the equivalent of superiority. If providers can achieve the same clinical outcome with a 5-day course of antimicrobials as with a 7-day course, then 5 days is better. There is a tremendous volume of data, be it animal models, translational, or clinical, that associates prolonged antibiotic use with adverse long-term outcomes. Every day of additional antimicrobial therapy impacts the neonate’s microbiomes—gut, lung, skin, and more—leading to increased risk for multi-drug-resistant organism colonization and infection, candidiasis, NEC, late-onset sepsis, and death. In addition, the normal microbiome has the benefit of being anti-inflammatory; creating dysbiosis leads to more inflammation in the gut (NEC, malabsorption, extra-uterine growth failure); the lung (increased risk for bronchopulmonary dysplasia or asthma); the skin (increased risk for eczema) [53]. The risk of metabolic diseases such as obesity, metabolic syndrome, and diabetes also increases. The primary risk factor for all these observations is duration of therapy. Every day beyond the minimum duration necessary is going to raise these risks without providing additional short-term clinical benefit. Therefore, if 4 days is just as good as 7 days for neonatal pneumonia, then 4 days should be viewed as superior to 7 days.

Second, we should be wary of large, one-size-fits all answers. The devil is in the details when it comes to optimal therapy. Host factors (e.g., degree of prematurity, mechanical support), geographic factors (e.g., level IV versus level II, inborn versus referral NICUs, developed versus developing nations), and microbe factors (e.g., *S. aureus* behaves differently than *E. coli*, which behaves differently than *Pseudomonas*, etc.) all play a role in deciding duration of treatment. However, by over-focusing on these specific factors, investigators risk undermining generalizability. Would it be preferable to have a single, large randomized controlled trial of 1000 preterm infants comparing 3 days versus 7 for UTI? Or would it be preferable to have four similar trials of 250 preterm infants each—one focusing on <1000 g infants, one focusing on term infants, one focusing on infection with *E. coli* only, and one focusing only on moderately preterm infants with abnormal renal ultrasounds? This tug-of-war between generalizability and internal validity is a question in search of answers; until then, clinicians should be appropriately skeptical of over-generalizing findings from one study to their patients if the clinical situation is significantly different.

Third, clinical trials need to be large enough to be adequately powered and the investigated difference in therapy needs to be clinically relevant (e.g., at least 3–4 days, or 20% of total duration). The effort required to shepherd a randomized controlled trial across the finish line is substantial; investigating the difference between 14 days and 12 days of therapy is a waste of that effort. On the other hand, the difference cannot be recklessly large [54]. A randomized controlled trial of 21 days versus 3 days of therapy for stage III NEC would be unethical and would not make it past institutional review. Small but clinically meaningful reductions should be studied; once a new safe, effective duration is identified, then that can provide a starting point for the next attempt at reduction (e.g., 14 days → 10 → 7 → 5 → 3 → 2, etc., until the shorter duration proves to be inferior). If we are approaching the cliff edge blindfolded, we should find the edge by tiptoeing up to it, not sprinting off it.

Finally, clinical trials do not need to set an *a priori* duration of therapy; instead, trials can compare traditional, dogmatic durations to “until clinically improved.” [55]. Once objective clinical criteria are achieved, antibiotics can be discontinued. For example, the DurATi-n study [56] in Denmark aims to enroll 488 infants ≥35 weeks gestation with “culture-negative” early-onset sepsis; infants are randomized 1:1 to 7 days of antibiotics versus discontinuing antibiotics once they are clinically well-appearing and have a C-reactive protein ≤ 30 mg/L. By utilizing individual clinical criteria, total duration of therapy may be safely shortened from the traditional 7 days. A similar approach combining clinical, laboratory, or radiographic findings could work well for other neonatal infections, including NEC, pneumonia, skin and soft tissue infections, and osteomyelitis. Challenges will remain, such as standardizing endpoint criteria for a given infection (e.g., resolution of respiratory distress for pneumonia, degree of improvement versus normalization of abdominal imaging for NEC) and ensuring safety guardrails are in place; however, reframing outcomes to patient-level success could garner support of clinicians and families alike.

## 6. Conclusions

Optimally, neonatologists would treat an infection until it has resolved, and not a moment longer. Finding this theoretical sweet spot is much easier said than done, however. Observational studies have hinted at safety and efficacy with shorter durations for some clinical scenarios, but virtually all these observational studies are subject to bias and confounding and are therefore hypothesis-generating at best. On top of that, there is a significant dearth of clinical trial evidence. At present, no trial has shown the superiority of longer treatment compared with traditional, dogmatic durations—that is to say, no one is arguing for 5 weeks of therapy for uncomplicated early-onset sepsis, or 2 months of therapy for a UTI—but there is certain to be a lower limit of duration that is too short to ensure ideal outcomes. This has already been demonstrated for neonatal pneumonia, where 2 days of therapy was inferior to 4 days in a small trial. This highlights that we must approach these lower limits of treatment duration carefully, with stepwise, ethical clinical trials that help us define how short is too short. These studies must consider individual circumstances; one-size-fits-all durations should be viewed with caution. Most likely, studies that focus on individualized therapy—infant-specific clinical responses guiding treatment duration for that infant—will be superior to blanket statements regarding a single optimal number for a given condition. If we approach each infection with consideration; if we carefully and cautiously chisel away the unnecessary, the prolonged, the superfluous; if we can leave only the antibiotic doses that are truly necessary—what remains will be a work of art. Together, we can free the angels.

## Figures and Tables

**Table 1:** Conceptual framework for designing clinical studies for optimal antibiotic duration in neonatal infections.

Concept	Examples	Notes
Primary endpoint	Late-onset *E. coli* sepsis	
Duration-based	14 days versus 7 days	Duration-based is traditional, but clinically based durations may better inform patient-level stewardship efforts.
Clinical outcome-based	14 days versus “until abdominal examination and radiography normalizing”	
Microbiology-based	14 days versus 7 days from first sterile culture	
Laboratory-based	14 days versus “until C-reactive protein is <20 mg/L”	
		
Intra-trial safety methodology		
Specific rescue criteria	e.g., specific clinical or laboratory criteria for broadening or extending therapy	Clear rescue criteria and safety monitoring will promote both patient safety and physician willingness to participate in trial. Specificity will also support meta-analysis of duration studies across centers.
Minimum safety monitoring	Therapeutic drug levels, serum testing for acute kidney injury, hepatic injury, or other drug sequelae as appropriate	
Specific criteria for re-evaluation	e.g., standardizing number of radiographs, laboratory testing, or cultures to ensure clinical resolution	
		
Primary marker(s) of failure		Time frame will depend on clinical indication; for necrotizing enterocolitis or pneumonia, 1–2 weeks may be logical; for meningitis or osteomyelitis, a longer window is logical.
Recurrence within a set time period	e.g., recurrence of pneumonia within 7 days of stopping therapy	
Another infection with same organism within a set time period	e.g., group B streptococcal (GBS) urinary tract infection 11 days after completing therapy for GBS bloodstream infection	
Mortality		
		
Secondary markers of safety		
Mortality		Partnering with pharmacologists remains critical for duration of therapy trials.
Antimicrobial resistance	e.g., subsequent colonization with extended-spectrum beta-lactamase producing organism	
Antibiotic-associated sequelae	e.g., acute kidney injury from gentamicin or vancomycin	

## Data Availability

No new data were created or analyzed in this study. Data sharing is not applicable to this article.

## Abbreviations

NANO	NICU Antibiotics and Outcomes
NEC	Necrotizing enterocolitis
NICU	Neonatal intensive care unit
UTI	Urinary tract infection
